# Habitat suitability assessment of mouflon applying a comparative approach on expert-based and species distribution models

**DOI:** 10.1371/journal.pone.0353063

**Published:** 2026-09-21

**Authors:** Azita Rezvani, Shekoufeh Nematollahi, Davoud Fadakar, Sedighe Abdollahi, Sima Fakheran

**Affiliations:** 1 Department of Natural Resources, Isfahan University of Technology, Isfahan, Iran; 2 Department of Environment, Faculty of Natural Resources and Environment, Ferdowsi University of Mashhad, Mashhad, Iran; 3 Faculty of Forestry, University of British Columbia, Vancouver, Canada; The University of Auckland - City Campus: University of Auckland, NEW ZEALAND

## Abstract

During the last decades, anthropogenic activities such as land use change and urbanization have caused significant biodiversity loss and ecosystem services disruption worldwide. Accurate identification and prioritization of suitable habitats, both within and beyond existing protected areas and ecological networks, require complementary habitat assessment models that evaluate species-specific suitability alongside habitat conservation priority. This study employed the maximum entropy (MaxEnt) and Integrated Valuation of Ecosystem Services and Tradeoffs (InVEST) models to assess the habitat of mouflon (*Ovis gmelini*) in central Iran. We specifically investigated how these models, with their distinct data inputs—MaxEnt relying on species occurrence and environmental variables, and InVEST utilizing land use/cover maps, and expert-based threat assessments—provide complementary insights for detecting and managing suitable habitats. Model performance was evaluated using the Area Under the Curve (AUC) of the Receiver Operating Characteristic (ROC) curve and the Boyce Index (BI). The spatial outputs were further analyzed using the Getis-Ord Gi* statistic to identify statistically significant hotspots. The results indicated that MaxEnt was highly effective in identifying currently occupied (realized) suitable habitats within protected areas (AUC = 0.937, BI = 0.965), reflecting areas that are already being used by mouflon populations, whereas InVEST identified broader extents of potential habitats, including areas that are ecological suitable, but are not consistently or currently utilized by the species. The spatial overlap between models was only 7.3% within protected areas, underscoring their complementary nature. Hotspot analysis revealed that while areas of high habitat quality and high suitability overlapped in some core habitats, their spatial distributions also showed distinct differences. For enhancing conservation networks under data-poor conditions, InVEST offers valuable guidance by highlighting priority zones based on habitat quality and degradation. However, the combined application of both models provides a robust framework for conservation planning, enabling the identification of core habitats, connectivity corridors, and degraded areas requiring restoration. These findings are critical for conserving wide-ranging ungulates like the mouflon and support the development of resilient ecological networks in the face of anthropogenic and climate change pressures.

## Introduction

Anthropogenic threats are increasingly impacting ecosystems and habitats worldwide, resulting in widespread habitat degradation and significant biodiversity loss [1,2]. Drivers such as human population growth, land use changes, and infrastructure development have escalated the extinction risk of many species, particularly large mammals [3–5]. In light of the challenges faced by arid and semi-arid ecosystems, it is essential to identify areas of high habitat value, prioritize conservation efforts, and recognize gaps through ecosystem service network [6,7].

Maintaining biodiversity in these vulnerable environments requires planning approaches that integrate ecological, social, and spatial dimensions. The growing urgency to protect biodiversity and associated ecosystem services within and around designated conservation areas has become evident [8]. Persistent habitat loss and fragmentation in drylands highlight the importance of strategies that improve habitat quality, strengthen ecological integrity, and enhance connectivity among habitat patches [7,9]. Furthermore, climate change presents an escalating threat, indicating substantial habitat loss and range shifts for mountain ungulates in Iran, potentially reducing the efficacy of existing protected areas [5,10]. A collaborative conservation framework can therefore help mitigate the adverse effects of human activities while sustaining essential ecosystem functions and services [11].

Protected areas have been established as core conservation strategies, supported by systematic conservation planning tools such as Marxan [12], C-Plan [13], and ConsNet [14], which help planners determine the optimal location and configuration of conservation areas to maximize biodiversity value. In recent years, conservation efforts have increasingly employed species distribution models (SDMs) to analyze the relationship between environmental factors and biodiversity [15]. These models, as discussed by Velasco-Rodríguez et al. (2025), provide valuable tools for predicting species distributions under varying environmental and anthropogenic scenarios [16]. The InVEST model represents one such tool designed to assess habitat quality by integrating data on anthropogenic threats, land use/land cover (LULC), and expert knowledge [17]. Although originally developed for ecosystem-scale assessments, InVEST can be adapted to species-level analysis when the selected species is strongly linked to habitat condition, as is the case for large herbivores like the mouflon. This adaptation allows the model to capture spatial patterns of habitat degradation and conservation value relevant to the species. InVEST evaluates habitat condition and provides reliable estimates of biodiversity response to threats, operating under the assumption that higher habitat quality supports greater native species richness [2,18–21], and notably does not require prior data on species presence or distribution.

Habitat quality is defined by the extent to which a habitat supports species survival and ecosystem stability, influenced by factors such as habitat structure, vegetation, food sources, water availability, and human threats [22]. In contrast, habitat suitability measures the likelihood of a species’ presence based on environmental and ecological conditions [23]. This principle is operationalized in SDMs like MaxEnt, which analyzes species occurrence records alongside environmental variables to predict the realized niche, facilitating local-level analyses and identifying areas suitable for specific species [24].

Large herbivores often act as flagship and umbrella species in arid and semi-arid ecosystems, where their presence indicates ecological integrity and supports multiple ecosystem services, including vegetation structure maintenance, nutrient cycling, and habitat provision for other species. In this context, the mouflon (*Ovis gmelini*) represents a key species whose population trends reflect both habitat quality and the cumulative effects of anthropogenic pressures. Mountain ungulates like the mouflon are particularly vulnerable to environmental changes, as they often inhabit specific elevational ranges with limited options for upward migration [5,25]. Therefore, assessing its habitat suitability and quality can provide valuable insights for broader conservation planning and ecosystem service management in central Iran.

In Iran, the mouflon (*Ovis gmelini*) and the urial wild sheep (*Ovis vignei*) overlap in range, with their hybrids exhibiting intermediate chromosome numbers [26]. Rezaei et al. (2010) confirmed their distinct taxonomic status through mtDNA analysis [27]. The mouflon is a flagship ungulate species of mountainous and arid landscapes in central Iran, and its conservation is vital for maintaining the structure and function of these ecosystems, as it influences vegetation dynamics and serves as prey for large carnivores. This wild sheep species is highly adaptable to rugged and arid landscapes, preferring open grasslands and hilly terrain with access to water and forage. Its distribution is influenced by both natural and anthropogenic factors, with suitable vegetation playing a critical role in habitat selection since the mouflon primarily feeds on grasses, shrubs, and forbs. Steep slopes and rocky terrain also provide natural shelter from predators [5,28].

Mouflon forms large herds within protected areas in central Iran, such as Ghamishloo National Park and Mooteh Wildlife Refuge, and exhibits seasonal migration between summer and winter habitats [29]. Historically distributed across western Iran from Hormozgan and Kerman in the south to West Azerbaijan in the northwest, the mouflon now faces increasing threats from intensive hunting, habitat degradation, and competition with livestock, leading to significant reductions in population sizes and distribution ranges [30,31]. Despite these threats, populations persist around protected areas, though habitat fragmentation from infrastructure expansion and human settlements raises the risk of isolation. The mouflon is currently classified as Near Threatened (NT) on the IUCN Red List [31].

This study evaluates how the InVEST and MaxEnt models can be used synergistically rather than comparatively to provide a comprehensive understanding of mouflon habitat. MaxEnt identifies suitable habitats based on occurrence data, while InVEST highlights habitat quality and degradation patterns across the landscape. By integrating the outputs of both models and applying a Getis-Ord Gi* hotspot analysis, this study offers a spatially robust framework for conservation decision-making, particularly for identifying core habitats, priority conservation areas, and potential migration corridors in central Iran.

## Materials and methods

### Study area

**Fig 1:**
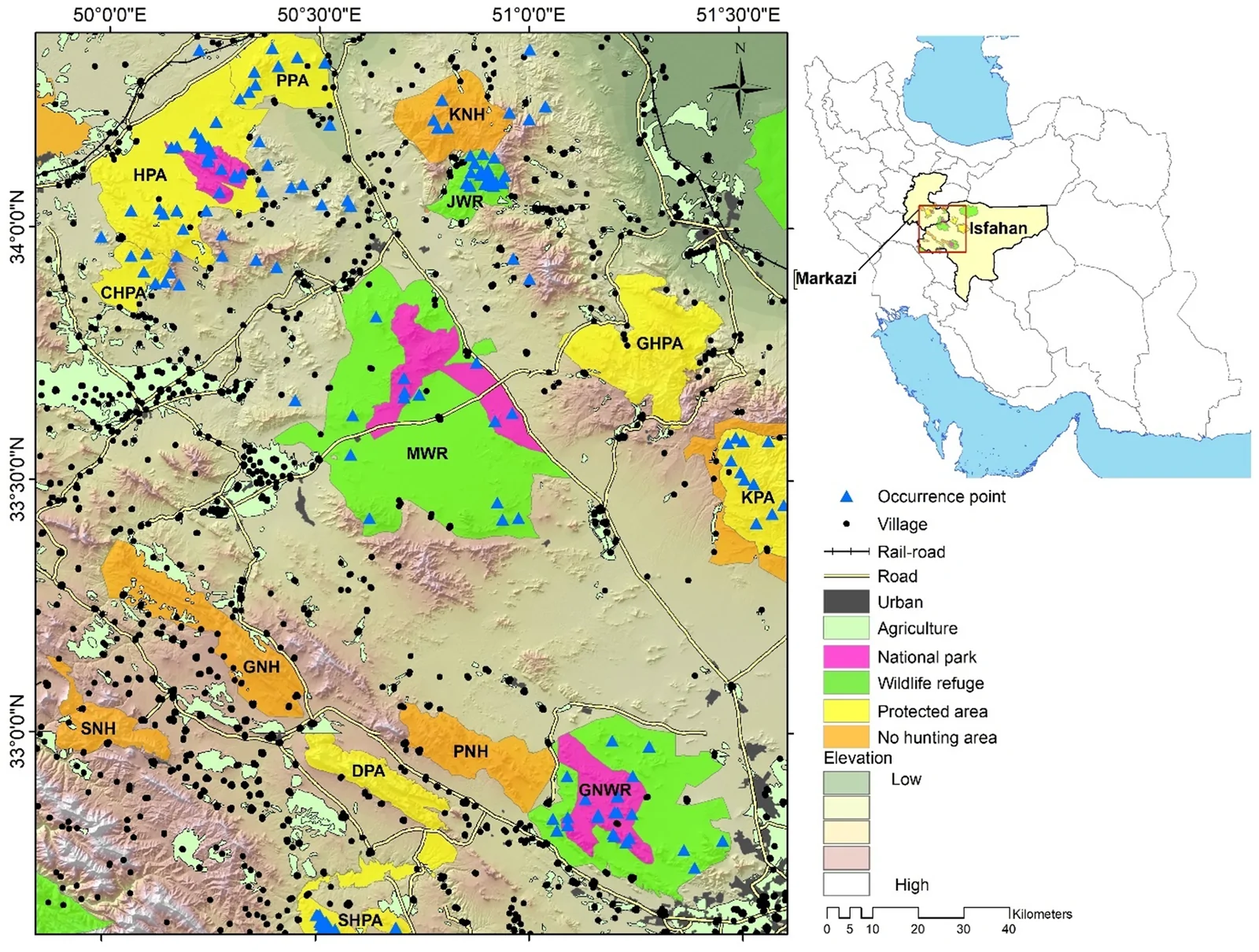
Location of the study area and protected areas in Isfahan and Markazi provinces in central Iran. The full forms of abbreviations used in this figure are provided in Table 1.

The study was conducted in 14 key habitats of the mouflon in central Iran covering a total area of approximately 3,850 km² (Fig 1, Table 1). Collectively, these areas encompass mountainous landscapes, hills, and plains, with an overall elevation range of approximately 1,580–3,000 m above sea level.

**Table 1:** List of protected areas within the study region.

Name	Abbreviation
Haftad Gholleh Protected Area	HPA
Mooteh Wildlife Refuge	MWR
Ghamishloo National Park and Wildlife Refuge	GNWR
Kahak No Hunting Area	KNH
Jasb Wildlife Refuge	JWR
Karkas Protected Area	KPA
Sheida Protected Area	SHPA
Ghamsar and Barzak Protected Area	GHPA
Palang Galoun No Hunting Area	PNH
Golestan Kooh No Hunting Area	GNH
Setableh No Hunting Area	SNH
Dalankooh Protected Area	DPA
Palang-Dareh Protected Area	PPA
Chenar Protected Area	CHPA

The climate across the study area is predominantly semi-arid to cold semi-arid, characterized by hot, dry summers and cold winters. Recorded temperatures range from approximately −18.5°C to 41.5°C, and the mean annual precipitation varies between 180 and 190 mm [32]. In most parts of the region, permanent rivers are absent; therefore, water availability for wildlife depends primarily on springs, stone troughs, and vegetation moisture [33].

Vegetation is mainly composed of cold steppe and dry shrubland communities. The dominant plant taxa include species of *Artemisia* spp. and *Astragalus* spp., along with other drought-tolerant shrub-steppe species from the Asteraceae, Brassicaceae, and Poaceae families [32–34]. Shrublands are particularly characterized by *Artemisia sieberi* and *Artemisia aucheri*, accompanied by genera such as *Cousinia*, *Euphorbia*, and *Scariola* [33]. These habitats provide essential ecological conditions for mouflon (*Ovis gmelini*) populations across the study area. However, the region is increasingly affected by anthropogenic pressures, including mining activities, industrial development, road expansion, and residential growth.

### InVEST modelling for habitat quality

In this section, the habitat quality module of InVEST was applied to map the habitat quality (HQ) and habitat degradation (HD) of the study region [17,35]. The main input layers for the habitat quality module of InVEST included the LULC map of the study area and anthropogenic threats affecting the species. These layers were obtained from the Iranian Department of the Environment (DoE) and the Iranian Forests, Range, and Watershed Management Organization (FRWO). The LULC map was prepared at a scale of 1:50,000.

To evaluate and quantify mouflon habitat quality, we used InVEST v.3.4.4, which models LULC as production functions using simple biophysical models. The InVEST model estimates habitat quality based on three key factors: (1) The suitability of each LULC type (see Supporting information, S1 Fig) in providing habitat for biodiversity. (2) The impact of various anthropogenic threats on habitat quality such as distance to roads, villages, urban areas, mines, and agricultural areas. (3) The sensitivity of each LULC type to each identified threat.

A relative habitat suitability score (*Hj*), ranging from 0 to 1 with 1 representing the highest suitability, was assigned to each habitat type. A key feature of the InVEST model is its ability to specify the sensitivity of habitat types to different threats, recognizing that not all habitat types are affected in the same way. Each threat was mapped as a raster layer, where the grid cell value, normalized between 0 and 1, represents the threat intensity within that cell.

The impact of threats on habitat quality was determined by three key factors including the distance from the threat source, defined as Max.D, which is the maximum distance a threat can influence habitat quality, the relative weight of each threat, represented as Wr, which indicates its importance compared to other threats on a scale from 0 to 1, and the sensitivity of each habitat type to the threat, referred to as *Sjr*, which determines how strongly the habitat degrades in response to the threat.

In general, threat impact decreases with distance from the degradation source. Cells closer to the threat experience higher impacts, while those beyond Max.D remain unaffected. Additionally, threats vary in their destructive impact on different habitats, and habitat types with higher *Sjr* values are assumed to degrade more severely.

For this case study, threat parameters and *Hj* values were initially determined based on expert knowledge. Since this module is sensitive to expert input, we consulted fifteen specialists with diverse backgrounds, including ecological modelling and experimental ecology, to assign values to the model parameters. Before scoring, experts were provided with a detailed explanation of the parameters they were asked to evaluate, the functioning of the habitat quality model, and the structure and interpretation of the tables they needed to complete (S1 and S2 Tables).

To reduce bias, a weighted averaging method was used for expert opinions, and invalid responses were eliminated. The final averaged scores from the valid questionnaires were then obtained using the ‘psych’ package in R software to model habitat quality.

### MaxEnt modelling for habitat suitability

#### Species presence data.

Mouflon occurrence data were obtained from direct observations, identification of footprint patterns, and feces, with the assistance of game wardens between 2020–2024. Geographic coordinates were recorded using handheld GPS devices (WGS84 datum; positional accuracy ±5 m). A total of 130 occurrence points were collected during field surveys, ensuring broad spatial coverage across the study area. In addition, 42 occurrence records were retrieved from the Global Biodiversity Information Facility (GBIF) and the DoE.

All occurrence records were initially examined for positional accuracy and ecological plausibility using high-resolution topographic layers in ArcGIS v.10.3 and Google Earth. Records with obvious spatial errors or implausible habitat locations were excluded. To reduce potential bias associated with human-dominated landscapes, a 10 km exclusion buffer was generated around settlements [36]. Presence points located within these buffers were removed after confirming that no major anthropogenic land-use changes occurred within the buffered areas during the study period. Spatial autocorrelation in the filtered dataset was assessed using Moran’s I in ArcGIS v.10.3 [37] to quantify the degree of spatial dependence among occurrence records. Subsequently, duplicate records (identical geographic coordinates) were removed in R v.4.3.1 (R Core Team, 2023) using the dplyr package. To further reduce spatial clustering and sampling bias, a 1 km spatial thinning procedure was implemented using the spThin package, retaining a single occurrence record within each 1 km radius. After applying all filtering and spatial rarefaction procedures, 168 spatially independent occurrence records were retained for subsequent modelling analyses. We followed the ODMAP (Overview, Data, Model, Assessment, and Prediction) protocol to ensure transparent, reproducible, and systematic reporting of all modelling steps, in accordance with established guidelines [38,39] (S3 Table). This study focused exclusively on species distribution modelling and did not involve animal handling, experimentation, genetic analyses, or invasive procedures. Ethical approval was granted by the Educational Office of the Department of Natural Resources, Isfahan University of Technology, Iran (letter number code 266, dated 02 September, 2020).

#### Environmental variables and selection.

For habitat suitability modelling, we compiled four groups of environmental predictors—bioclimatic, topographical, human activity, and habitat-related variables—based on mouflon ecology and published literature. Climatic variables were initially obtained from the CHELSA database at a ~ 30 arc seconds resolution (https://chelsa-climate.org). To downscale annual mean temperature and precipitation from coarse to fine resolution, a moving window regression approach was applied, using elevation as a covariate. Model performance was evaluated against observational data from synoptic and climatological stations (number of stations: 25; period: 1980–2024) provided by the National Meteorological Organization of Iran, with linear regression showing the highest accuracy. R² values indicate the proportion of variance explained by the model, and climatic variables were subsequently adjusted accordingly. Values of 19 bioclimatic variables and 1,000 pseudo-absence points were extracted, and multicollinearity was assessed using variance inflation factors (VIF) in R (usdm package; [40]). Variables with VIF > 10 were retained, resulting in five predictors for species distribution modeling: mean diurnal range, isothermality, precipitation seasonality, precipitation of warmest quarter, and precipitation of coldest quarter.

Topographical variables were derived from a 30m resolution digital elevation model (DEM) from the National Cartographic Center of Iran, including slope, categorical aspect, and terrain roughness using Surface Analysis tools in ArcGIS v.10.3. Human activity variables—distance to roads, villages, urban areas, mines, orchards, and agricultural areas—were extracted from 1:50,000 topographic maps and subsequently updated using high-resolution Google Earth imagery.

Habitat-related variables included distance to rivers, water sources, dams, and dominant vegetation types relevant to mouflon ecology (e.g., *Artemisia* spp., *Astragalus* spp., *Anabasis* spp., and *Scariola* spp.), obtained from the FRWO. All environmental predictors were resampled to a 30m spatial resolution to match the resolution used in the InVEST model.

To reduce multi collinearity, we calculated the VIF using the usdm package in R [40], and variables with VIF > 10 were excluded from further analyses. Remaining predictors were evaluated based on their contribution to habitat suitability modeling, while highly correlated variables were removed prior to contribution assessment to improve model transferability. Ultimately, elevation, slope, aspect, distance to agricultural and urban areas, distance to *Anabasis* spp. and *Scariola* spp., along with isothermality, were included as predictors in the habitat suitability modeling process. All retained environmental layers were converted to ASCII format for input into MaxEnt for habitat suitability modelling.

#### Habitat suitability modelling.

MaxEnt is a machine learning algorithm that requires only presence data and was executed in MaxEnt software v.3.3 [41]. This model offers advantages over other algorithms, such as the ability to predict species distributions using only presence data [41] and to function effectively with small sample sizes [42]. We used presence data along with environmental layers to model habitat suitability.

In the modelling process, 75% of the occurrence data were used for training, while the remaining 25% were reserved for testing. The MaxEnt model was run with 10 replicates using the subsample method, with maximum model iterations set to 500. The number of background points was set at 5,000, randomly selected as background points [28,43,44]. The logistic output format was chosen for the final results [42].

To evaluate model performance, we used the AUC of the ROC [45]. AUC measures the model’s ability to distinguish between suitable and unsuitable habitats, with values ranging from 0.0 to 1.0. A score of 0.5 indicates random prediction, while values greater than 0.9 indicate high predictive accuracy [10,41]. In addition to AUC, we validated the model using the BI [46] in the ecospat package [47]. The BI values range from −1–1, where −1 indicates a poor model, 0 represents a random model, and 1 indicates a perfectly predictive model; it measures how much model predictions differ from random distribution of the observed presences across the prediction gradient [48]. We also examined the response curves of the top contributing variables to interpret their ecological relationship with mouflon presence.

The suitability and quality habitat maps were converted into binary habitat maps based on the 10th percentile training presence threshold. This threshold was chosen to exclude marginal areas with very low suitability while addressing spatial bias and uncertainties linked to outlier presence points [49]. The overlap between the binary habitat suitability and quality maps was then analyzed to identify shared habitats.

#### Mapping habitat hot spots.

Following the preparation of the habitat suitability and quality maps, the Getis-Ord Gi* method was employed in ArcGIS v.10.3 to identify habitat patches for the mouflon species within the study area. This approach calculates the Z-statistic, p-values, and confidence levels of habitat patches, allowing for the identification of spatial clusters with statistically significant high (hot spots) and low values using ArcGIS v.10.3 for spatial statistics (cold spots) [50].

To validate the results, ROC curves were generated using the R software and the ‘pROC’ package. The ROC statistic was utilized to assess the spatial accuracy by comparing the habitat hot spot map to the species presence points, serving as a reference map.

## Results

### InVEST modelling for habitat quality

**Fig 2:**
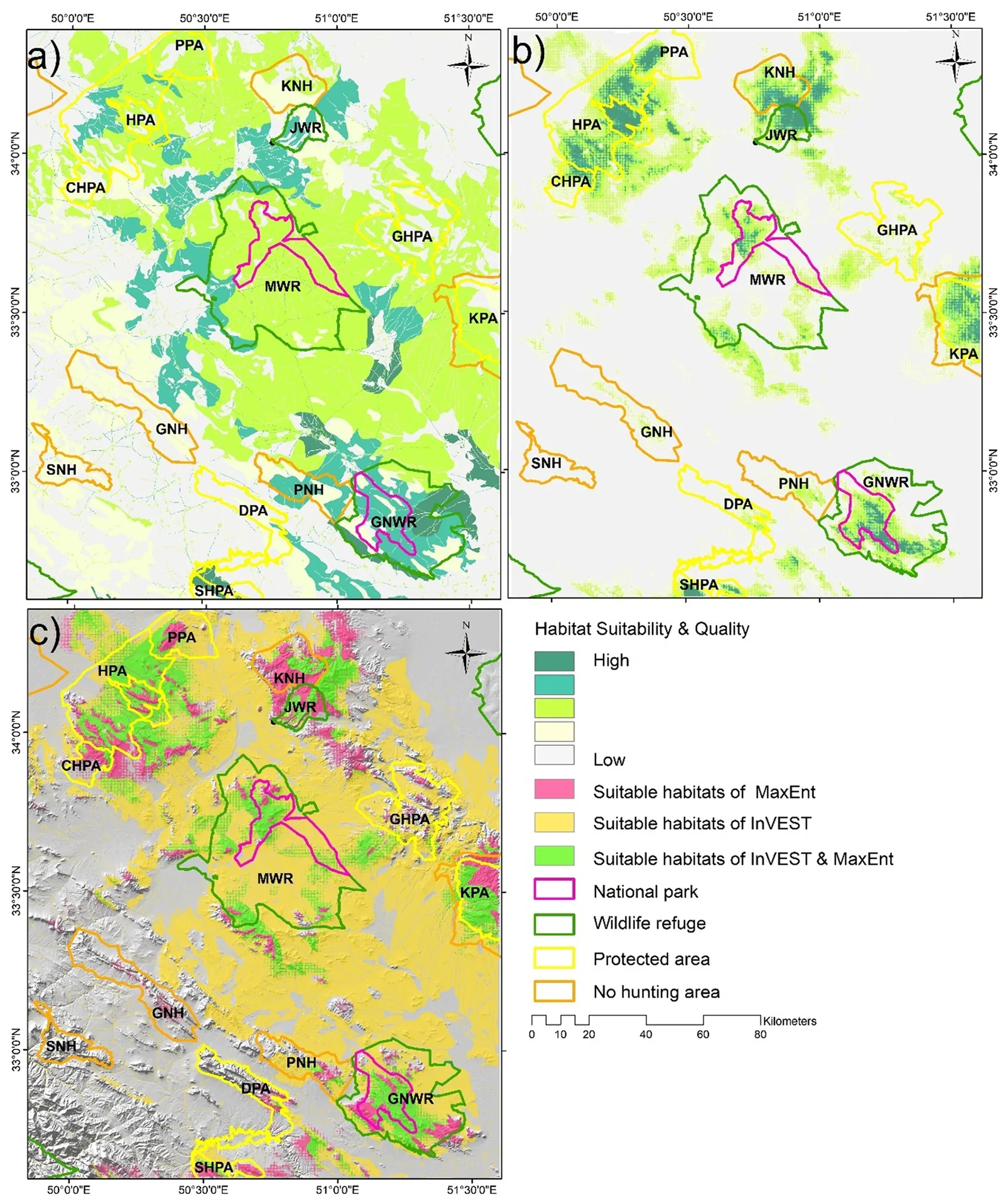
Spatial distribution of habitat quality for mouflon in the study area, with values ranging from 0 (lowest) to 1 (highest) (a). Habitat suitability map of mouflon produced using the MaxEnt model, with suitability values ranging from 0.0 to 1.0; gray (close to 0.0) denotes unsuitable areas, whereas green–blue (close to 1.0) indicates highly suitable habitats for species presence probability (b). Predicted habitat suitability of mouflon in central Iran derived from InVEST (orange), MaxEnt (pink), and their spatial overlap (green) (c).

HQ and HD maps showed distinct spatial patterns across the study area (Fig 2a, S2 Fig). HQ values, which ranged from 0 (lowest HQ) to 1 (highest HQ), were primarily higher in the northwest, southeast, and central sections of the study area (Fig 2a). On the other hand, the lowest HQ values were found near major settlement areas, which historically have been areas of high human activity, industrial development, and agricultural land use. These areas had a high density of anthropogenic threats and LULC, leading to low habitat quality. Overall, the InVEST HQ results indicated that approximately 45% of the study area provides suitable habitat quality for supporting mouflon populations, of which 86.7% is located within protected areas. The southern parts of the study area contained the highest proportion of high-quality habitats, highlighting their critical role in maintaining viable mouflon habitats and supporting conservation priorities (Fig 2a).

The HD values ranged from 0 (minimal human disturbance) to 0.11 (maximum human-induced degradation), with higher HD values concentrated around urban areas in the western and southwestern regions. These areas were characterized by dense road networks and high concentrations of industrial and settlement areas, further exacerbating habitat degradation. The HQ map indicated that the highest HQ values were linked to areas dominated by grassland species, such as *Scariola* spp. and *Astragalus* spp., which are vital for the mouflon.

### MaxEnt modelling for habitat suitability

The MaxEnt model was evaluated using the AUC of the ROC plot (AUC = 0.956) and the Boyce Index (BI = 0.971), both indicating that the model performed significantly better than random predictions. This suggests that the MaxEnt model has a high ability to predict the habitat suitability for mouflon in the study area.

The key predictors for determining habitat suitability included aspect, dem, distance from urban area, distance from vegetation types such as *Scariola* spp. and *Anabasis* spp., and distance from agricultural area (Table 2). Mouflon exhibited a strong association with areas close to preferred vegetation—particularly *Scariola* spp. (10.72%) and *Anabasis* spp.(9.29%)—as well as moderate elevations (31.24%) (S3 Fig). According to the habitat suitability map generated by the MaxEnt model (Fig 2b), extensive areas of highly suitable habitat are concentrated in the northern parts of the study area, largely within protected areas, particularly in PPA, CHPA, KNH, JWR, and HPA. In contrast, within the MWR, suitable habitats are mainly restricted to small and fragmented patches in the northern section of the refuge. In the southern parts of the study area, especially in SHPA and GNWR, the highest habitat suitability values were observed. Scattered and low-suitability patches occur in the central regions, indicating weak connectivity of suitable habitats in these areas. Overall, approximately 14% of the total study area exhibits high habitat suitability for supporting mouflon populations. Of these suitable habitats, about 89% are located within protected areas, while the remaining portion is mainly distributed in the surrounding buffer zones and peripheral areas of protected areas. This highlights the importance of these marginal habitats for conservation planning and the establishment of ecological corridors (Fig 2b).

**Table 2:** Relative contribution of environmental variables to the final MaxEnt habitat suitability model for mouflon in central Iran.

Variable	Percent contribution	Permutation importance
Aspect	31.24	1.07
Dem	16.02	32.49
Distance to urban	15.44	8.30
Distance to *Scariola* spp.	10.72	24.00
Distance to agricultural area	9.54	12.15
Distance to *Anabasis* spp.	9.29	12.14
Slope	5.75	1.25
Isothermality	2.01	8.60

### Model comparison: InVEST vs MaxEnt

The HQ map generated by InVEST, which evaluated the quality of each habitat type for mouflon based on LULC, anthropogenic threats, and expert knowledge, was compared with the habitat suitability map generated by MaxEnt, which utilized species occurrence data and environmental layers. Both models identified the northwest, southeast, and central sections of the study area as areas with high habitat suitability (Fig 2c).

The spatial overlap between suitable habitats predicted by the two models provided some important insights into their level of agreement and complementarity. MaxEnt predicted more suitable habitat (20.88%) within the protected areas compared to InVEST (14%), with a spatial overlap of 6.68%. Within protected areas, MaxEnt predicted a smaller proportion of suitable habitat (12.9%) compared to InVEST (39.5%), with an overlapping area of 7.3%. Around the protected areas, InVEST identified a larger extent of suitable habitat (6%) than MaxEnt (1.5%), with only 1.3% spatial overlap between the two models (Table 3, Fig 2c). Overall, the majority of suitable habitats were located within protected areas, particularly in areas with higher protection status, emphasizing the effectiveness of the current protected area network in conserving key mouflon habitats. In addition, similar suitable habitats were detected in mountainous landscapes surrounding protected areas, especially in the southern parts of HPA, the northern and southern margins of MWR, and the northern section of GNWR. These peripheral mountainous and hills habitats function as movement and dispersal corridors and likely serve as seasonal migration routes for mouflon, as supported by field observations.

**Table 3:** The proportion of suitable habitats for mouflon in central Iran.

	Within the protected areas		Around the protected areas	
	Area (km^2^)	Area (%)	Area (km^2^)	Area (%)
MaxEnt	4206.3	12.95	491.9	1.51
InVEST	12983	39.54	1979.4	13.23
Spatial overlap	2373.4	7.30	429.2	1.32

### Mapping habitat hot spots

**Fig 3:**
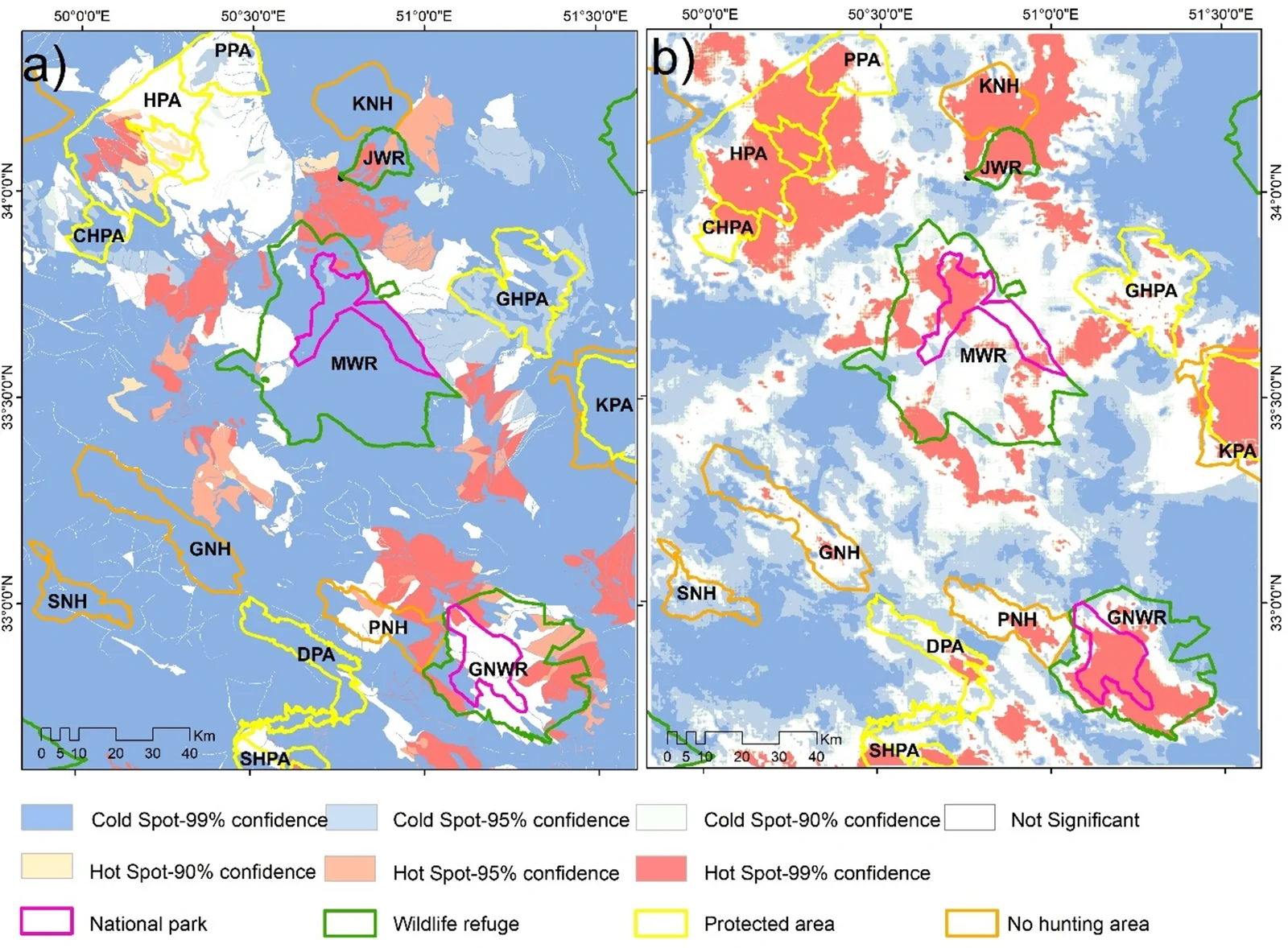
Spatial distribution of habitat hot spots and cold spots for the mouflon in central Iran. The left panel displays habitat hot spots generated by the InVEST model (a), while the right panel shows habitat hot spots produced by the MaxEnt model (b).

Fig 3 presents the results of the spatial statistical analysis of habitat patches derived from the MaxEnt and InVEST models. The figure reveals that habitat hot spots and high-value clusters exhibiting positive spatial correlations align with areas of maximum habitat suitability for the target species. Specifically, the InVEST model (Fig 3a) allocates 99% of the highest percentage (7%) and the largest area of habitat hot spots to these regions, while the MaxEnt model (Fig 3b) similarly designates 99% of the highest percentage (14.65%) and the largest area of habitat hot spots (Table 4).

**Table 4:** Area of habitat patches identified through Getis-Ord Gi* analysis.

	MaxEnt		InVEST	
	Area (ha)	Area (%)	Area (ha)	Area (%)
Cold Spot – 99% confidence	890120	27.40	2245026	71.29
Cold Spot – 95% confidence	729609	22.46	136617.8	4.34
Cold Spot – 90% confidence	223875	6.89	17467.47	0.55
Not Significant	841522	25.90	430676.7	13.68
Hot Spot – 90% confidence	32515	1.00	22303.49	0.71
Hot Spot – 95% confidence	55253	1.70	93204.74	2.96
Hot Spot – 99% confidence	475995	14.65	227973	7.24

Conversely, given the species’ range of occurrence within protected areas, a substantial portion of the study area is classified as habitat cold spots and regions exhibiting a low cluster pattern for the mouflon. Furthermore, the spatial accuracy and precision of the distribution pattern maps generated by the Getis-Ord Gi* method were assessed and compared using a performance characteristic diagram. The ROC reported for these models are 0.6 for the InVEST model and 0.91 for the MaxEnt model, indicating an acceptable efficiency for identifying mouflon habitat hot spots (S4 Fig).

## Discussion

This study demonstrates the value of integrating the InVEST and MaxEnt modelling frameworks to generate a multi-faceted understanding of habitat for the mouflon in central Iran. Our analysis reveals that while there is a partial spatial agreement between the models, their most significant contribution lies in their divergent outputs, which, when synthesized, provide a more complete picture for conservation planning.

### InVEST modelling for habitat quality

Key environmental factors influencing the presence of the mouflon, including altitude, slope, vegetation cover, distance to settlement points and roads, and water availability, were concentrated in high HQ areas [34,51]. Conversely, the lowest HQ values were identified near major settlement areas, which have historically experienced high human activity, industrial development, and agricultural land use. These locations exhibited a high density of human-related threats and LULC changes, resulting in degraded habitat quality. In contrast, the lowest HQ values were concentrated around the main settlement complexes in the western and northwestern sections of the study area. These zones have undergone intensive agricultural expansion, industrial development, and infrastructure growth over the past decades, resulting in pronounced habitat fragmentation. Correspondingly, the highest HD values were detected in the same urbanized regions, reflecting dense road networks and high levels of human disturbance. Overall, the spatial pattern of HQ and HD in our study area indicates that habitat degradation is strongly associated with localized anthropogenic pressures rather than uniform across the landscape. The spatial patterns of HQ and HD that we observed not only reflect the ecological preferences of mouflon but also highlight critical intersections with human-dominated landscapes. These intersections are not just important for species persistence, but also for ecosystem services — for example, water regulation, soil stability, and carbon sequestration — that are often reduced as habitat quality degrades. Recognizing these trade-offs can help inform land-management strategies that aim to restore or compensate for ecosystem services lost to urbanization and agriculture. For instance, establishing buffer zones or ecological corridors in areas of moderate HQ could both improve wildlife connectivity and maintain ecosystem functioning.

### MaxEnt modelling for habitat suitability

The MaxEnt model demonstrated robust predictive capabilities, as indicated by the high AUC and BI values. This performance underscores the model’s effectiveness in identifying suitable habitats based on species occurrence data and environmental variables [51]. As found in previous studies, anthropogenic factors play a crucial role in reducing the habitat security for mouflon [5,30]. Furthermore, the importance of topographic variables like slope aligns with known habitat preferences of the species for rugged terrain that offers refuge from predators and human disturbance [27,49].

### Model comparison: InVEST vs MaxEnt

The spatial overlap and divergence between the two models reveal critical, complementary insights for conservation strategies. While MaxEnt identified a more restricted extent of suitable habitats within protected areas, corresponding mainly to currently occupied (realized) habitats based on occurrence data, InVEST highlighted a broader distribution of potentially suitable habitats within and around these zones. The limited spatial overlap (6.68% within PAs, 1.13% around PAs) is not a model failure but a meaningful ecological signal. The areas of agreement, particularly within protected areas like GNWR, represent high-confidence core zones where high habitat suitability coincides with high habitat quality. These zones should be prioritized as critical areas for strict protection and management.

Conversely, the non-overlapping areas are equally informative. The suitable habitats identified by InVEST around protected areas are critical for the mouflon’s migratory patterns and potential range shifts. These areas can be interpreted as potential migration pathways and corridors, essential for connecting populations and facilitating movement in response to seasonal changes and long-term climate pressures [5,28]. For instance, the free areas between HPA and MWR, identified by both models, correspond to known migration routes that are currently vulnerable to habitat degradation and increasing anthropogenic pressures [28].

Furthermore, InVEST’s ability to map HD provides an actionable layer for managers. It pinpoints where otherwise suitable habitats (identified by either model) are under significant anthropogenic pressure. This allows for targeted conservation actions, such as working with local communities to mitigate specific threats (e.g., controlling livestock grazing, regulating road traffic) before these areas become completely unsuitable. This is a key advantage, moving beyond simply identifying where to protect, to understanding why a habitat is at risk and how to intervene.

This finding aligns with the notion that conservation efforts should extend beyond protected areas to encompass surrounding habitats that are essential for species survival, as emphasized by Hansen and Defries [52]. Both models identified the northwest, southeast, and central sections of the study area as areas with high habitat suitability. These regions were characterized by suitable environmental conditions such as appropriate vegetation cover, geographic features (elevation, slope), and proximity to water sources, which are essential for mouflon.

When comparing the results of both models, we observed that settlement areas, especially urban areas and road networks, were identified as the primary threats to the presence of mouflon in both models. The comparison revealed that both models had strong points that complemented each other.

MaxEnt, by utilizing topographic variables such as elevation and slope, provided valuable insights into habitat selection for mouflons [12,24]. However, its reliance on presence data means it may not fully capture the species’ use of lower-quality landscapes during migration [28]. As presence records are typically more abundant within protected areas, MaxEnt tends to focus on these regions, potentially overlooking important habitats around them. This presents a limitation when studying species like the mouflon, which migrate between protected and unprotected areas [28].

In contrast, InVEST complements this by providing a landscape-wide view of habitat potential and degradation, independent of presence points [53]. Since it relies on land use data and expert knowledge rather than presence points, it can effectively assess habitat quality across the entire landscape, including unprotected areas. This makes it especially valuable for identifying potential habitats and assessing the impact of anthropogenic threats where direct observations are scarce. In regions where species occurrence data are scarce, identifying suitable habitats presents a significant challenge. By using InVEST, conservation planners can gain insights into habitat suitability beyond the current protection network, supporting informed decision-making aimed at expanding conservation efforts and enhancing habitat connectivity for mouflons across management units.

One of the main advantages of MaxEnt is its ability to estimate species distribution based on occurrence points, increasing the model’s validity. However, collecting sufficient occurrence data can be challenging, which makes MaxEnt less feasible in certain contexts. In contrast, InVEST relies on expert opinions and LULC maps, making it a viable tool when field data is limited. InVEST also accounts for the impacts of anthropogenic threats by calculating the distance from threat sources, the relative weight of each threat, and the sensitivity of habitat types to those threats.

The overlap of habitat suitability areas between the two models showed some important insights. InVEST predicted more suitable habitat within the protected areas compared to MaxEnt, with a spatial overlap of 7.3%. Around the protected areas, MaxEnt predicted a larger area of suitable habitat than InVEST, with only a 1.3% overlap. The sensitivity of MaxEnt to occurrence points means that it may not adequately predict suitable habitats around protected areas if the species is mainly found within protected areas. However, suitable habitats around protected areas are critical for the species’ seasonal migration. Therefore, InVEST provides complementary information by predicting suitable habitats in these areas, which is important for expanding the protected area network.

The spatial distribution of habitat quality and suitability in both models demonstrated the importance of identifying new potential habitats, particularly around the current protected areas, to safeguard the long-term viability of mouflon populations. The combined use of MaxEnt and InVEST can minimize the risks of conservation actions by providing complementary information on habitat quality, degradation, and suitability.

### Mapping habitat hot spots

The identification of habitat hot spots provides valuable information for prioritizing conservation efforts. The clustering of high-quality habitats in specific regions suggests that targeted management strategies could enhance the effectiveness of conservation initiatives. In comparison to previous studies that have assessed and modeled the habitat suitability of the mouflon, this study specifically focused on spatial data mapping techniques to analyze suitable habitat areas and identify habitat hot spots for this species. The findings from the spatial data mapping evaluation indicated a significant impact of the mouflon habitat suitability map on the study area, revealing that the areas considered unsuitable for the species correspond to cold habitat zones.

Given that much of the study area consists of plains and urban development, a considerable portion is classified as unsuitable habitat for the mouflon. Only a small percentage of the area, particularly in and around protected area, demonstrates very high suitability, constituting the identified habitat hot spots. As shown in Fig 3a, the InVEST model reveals that a limited number of habitat hot spots with high clustering and a confidence level of 99% are located within protected areas, with hottest spots found around these zones where environmental threats are evident [54,55]. In contrast, the MaxEnt model identifies the optimal habitat regions for the mouflon primarily within protected areas [56], highlighting core population centers.

The outcomes of this study provide a valuable tool for enhancing conservation planning for the mouflon and similar species. By employing complementary models like InVEST and MaxEnt, particularly in circumstances with limited spatial data, conservation strategies can be more effectively developed to bolster the protection of threatened species. Furthermore, based on these results, the level of suitable habitat for the mouflon can be improved through targeted management and planning efforts addressing the key parameters influencing habitat suitability in the study area.

The complementary use of InVEST and MaxEnt models not only enhances the understanding of habitat dynamics but also informs decision-makers about the potential impacts of land use changes on wildlife habitats. As highlighted by Araújo and New (2007), integrating multiple modeling approaches can provide a more comprehensive assessment of habitat quality and suitability, ultimately leading to more effective conservation planning [57].

### Implications for multi-species conservation and climate change resilience

Both mouflon and goitered gazelle (Gazella subgutturosa) [37,58] are flagship species in central Iran, and historical migration has been recorded for both species between these areas [26]. Although this study was focused on the mouflon in central Iran, managers must consider these finding for conservation plans and designing corridors within the broader context of multi-species conservation and future climate change. It worth notice that while some part of the study area such as the plain areas in Mooteh Wildlife Refuge were not identified as suitable for the mountainous mouflon, they are known to be suitable for the plains-dwelling gazelle [10]. Therefore, an integrated conservation approach that overlays suitable habitats for both flagship species would provide managers with a powerful tool to design a cohesive protected area network. So, a multi-species perspective is crucial for building resilience to climate change.

Here, projections indicate severe habitat loss for mouflon (69–76% by 2070) and fragmentation of over 40% of its movement corridors [5,10]. In this scenario, the core habitats we identified (e.g., in GNWR) are likely to function as vital climatic refugia for mouflon and the corridors identified by InVEST around PAs will become increasingly important as pathways for forced range shifts for mouflon. But, designing conservation actions need to consider multiple, co-occurring ungulates (such as mouflon and goitered gazelle), so with this approach, managers will be able to create a connected landscape that facilitates gene flow of multiple species, especially flagship species and allows them to adaptively respond to both anthropogenic and climate pressures.

## Conclusion

This study used two complementary habitat modelling approaches, InVEST and MaxEnt, to assess the habitat quality and suitability of mouflon in central Iran. The InVEST model, based on LULC, anthropogenic threats, and expert knowledge, estimated habitat quality, while the MaxEnt model, based on species occurrence data and environmental layers, predicted habitat suitability. The spatial agreement in areas like GNWR helps identify robust core zones for strict protection. The spatial divergence, where InVEST identifies suitable habitat around protected areas, highlights potential migration pathways and corridors essential for connectivity.

The MaxEnt model accurately identified suitable habitats within protected areas, reflecting the species’ current distribution, whereas the InVEST model identified a broader distribution of potentially suitable habitats extending beyond these zones, highlighting areas under degradation pressure but with high inherent quality. InVEST enables spatially explicit assessments of habitat degradation and anthropogenic pressures outside the current conservation network. This capacity supports informed decision-making by highlighting additional areas of high conservation value, thereby facilitating the expansion of protected networks and improving habitat connectivity.

The combined application of both models provides a more robust foundation for conservation planning than either model alone. For managers, this integrated approach offers a clear framework: use MaxEnt to define core population areas, use InVEST to map connecting corridors and degradation threats, and use the overlap to validate the highest-priority zones. Furthermore, this approach should be expanded to a multi-species context. The study area supports both mouflon and goitered gazelle, with the plain areas in MWR being unsuitable for mouflon but critical for the goitered gazelle. By integrating habitat models for all flagship species, managers can design a connected network of core areas and corridors that supports biodiversity as a whole, ultimately enhancing the ecosystem’s ability to withstand anthropogenic and climate change pressures.

By combining expert knowledge with species occurrence data, this study demonstrates how complementary modelling frameworks can move beyond simple habitat maps to provide dynamic, management-ready insights for conservation planning. The findings emphasize the importance of considering both protected and non-protected areas through an integrated lens to safeguard the long-term viability of the mouflon and the ecological integrity of central Iran’s landscapes.

Overall, this study underscores the importance of utilizing advanced modeling techniques to assess habitat quality and suitability for the Asiatic mouflon. The findings advocate for targeted conservation strategies that address anthropogenic threats and prioritize the protection of critical habitats, particularly those identified as hot spots. By adopting a holistic approach that combines habitat quality assessments with species distribution modeling, conservation practitioners can better safeguard the long-term viability of the mouflon and other threatened species.

## Supporting information

S1 Table Mean values for habitat suitability (*Hj*) and the relative sensitivity of habitat types to threats (*Sjr*) obtained from expert knowledge and literature review and subsequently adjusted during the calibration of the habitat quality model using empirical biodiversity data (Sallustio et al. 2017; Terrado et al. 2016). (PDF)

S2 Table Characteristics of threat sources required for the InVEST habitat quality model. *Wr* and *Max.D* refer to the mean values of weights and maximum distance to threats which affected habitat quality obtained from expert knowledge and literature review (Sallustio et al. 2017; Terrado et al. 2016).
(PDF)

S3 Table ODMAP summary of the modelling workflow, documenting all steps of data preparation, model building, evaluation, and prediction for the habitat suitability analysis. (PDF)

S1 Fig The land use and land cover map of the study area based on data from topography map (1/50000) obtained from FRWO and DoE. Land uses were aggregated into 17 different main categories corresponding to habitat types.
(PDF)

S2 Fig The degradation map of mouflon in the study area. Habitat quality value ranges between 0 (lowest) and 1 (highest) and habitat degradation is between 0 (minimum) and 0.11 (maximum of threats concentration).
(PDF)

S3 Fig Response curves showing the effect of key environmental predictors on mouflon habitat suitability in the MaxEnt model. (PDF)

S4 Fig Performance characteristic diagrams illustrating habitat hot spots. The left panel displays the results from the MaxEnt model, while the right panel presents findings from the InVEST model.
(PDF)
