# Supplementary material for: Habitat suitability assessment of mouflon applying a comparative approach on expert-based and species distribution models
Source: PLoS One. 2026 Sep 21;21(9):e0353063. doi: 10.1371/journal.pone.0353063 (PMC13592577; doi:10.1371/journal.pone.0353063)
Supplement: Supplementary file 1 [file pone.0353063.s001.pdf]

Supporting information

S1 Table. Mean values for habitat suitability (*Hj*) and the relative sensitivity of habitat types to threats (*Sjr*) obtained from expert knowledge and literature review and subsequently adjusted during the calibration of the habitat quality model using empirical biodiversity data (Sallustio et al. 2017; Terrado et al. 2016).

	**Habitat Type**	***Hj* [0-1]**	**Relative sensitivity of habitat types to threats (*Sjr*)**								
			**Urban**	**City**	**Village**	**Mine**	**First class**	**Second class**	**Third class**	**Agriculture**	**Dry farming**
1	**Urban (big city)**	0	0	0	0	0	0	0	0	0	0
2	**City**	0	0	0	0	0	0	0	0	0	0
3	**Village**	0.2	0.7	0.6	0	0.6	0.5	0.4	0.3	0.4	0.3
4	**Mine**	0	0	0	0	0	0	0	0	0	0
5	**First class (road)**	0	0	0	0	0	0	0	0	0	0
6	**Second class (road)**	0	0	0	0	0	0	0	0	0	0
7	**Third class (road)**	0.2	0.5	0.4	0.3	0.5	0.4	0.3	0	0.3	0.2
8	**Permanent river**	0.4	0.8	0.7	0.6	0.8	0.7	0.6	0.4	0.6	0.4
9	**Seasonal river**	0.3	0.7	0.6	0.5	0.7	0.6	0.5	0.3	0.3	0.2
11	**Agriculture**	0.33	0.6	0.5	0.3	0.6	0.5	0.4	0.3	0	0
12	**Dry farming**	0.36	0.6	0.5	0.4	0.5	0.5	0.4	0.3	0	0
13	***Scariola* spp.**	1	0.9	0.8	0.7	0.8	0.8	0.7	0.6	0.7	0.6
14	***Astragalus* spp.**	0.8	0.8	0.7	0.6	0.7	0.7	0.6	0.5	0.6	0.5
15	**No range**	0.2	0.6	0.5	0.4	0.5	0.5	0.4	0.3	0.5	0.4
16	**Outcrops**	0	0	0	0	0	0	0	0	0	0
