# Supplementary material for: Habitat suitability assessment of mouflon applying a comparative approach on expert-based and species distribution models
Source: PLoS One. 2026 Sep 21;21(9):e0353063. doi: 10.1371/journal.pone.0353063 (PMC13592577; doi:10.1371/journal.pone.0353063)
Supplement: Supplementary file 2 [file pone.0353063.s002.pdf]

Supporting information

S2 Table. Characteristics of threat sources required for the InVEST habitat quality model. *Wr* and *Max.D* refer to the mean values of weights and maximum distance to threats which affected habitat quality obtained from expert knowledge and literature review (Sallustio et al. 2017; Terrado et al. 2016).

**Threat**	***Wr* [0-1]**	***Max.D* (km)**	**Decay**
**Urban (big city)**	1	8	exponential
**City**	0.8	6.5	exponential
**Village**	0.6	5	exponential
**Mine**	0.8	6	exponential
**First class**	0.8	3	exponential
**Second class**	0.67	2	exponential
**Third class**	0.4	1	exponential
**Agriculture**	0.6	4	exponential
**Dry farming**	0.5	2.8	exponential
