# Supplementary material for: Habitat suitability assessment of mouflon applying a comparative approach on expert-based and species distribution models
Source: PLoS One. 2026 Sep 21;21(9):e0353063. doi: 10.1371/journal.pone.0353063 (PMC13592577; doi:10.1371/journal.pone.0353063)
Supplement: Supplementary file 3 [file pone.0353063.s003.pdf]

Supporting information

S3 Table. ODMAP summary of the modelling workflow, documenting all steps of data preparation, model building, evaluation, and prediction for the habitat suitability analysis.

section	subsection	element	Value
Overview	Authorship	Study title	Habitat suitability assessment of mouflon applying a comparative approach on expert-based and species distribution models
Overview	Authorship	Author names	Azita Rezvani; Shekoufeh Nematollahi; Davoud Fadakar; Sedighe Abdollahi; Sima Fakheran
Overview	Authorship	Contact	fakheran@iut.ac.ir; Azita.rezvani@na.iut.ac.ir
Overview	Model objective	Model objective	Forecast
Overview	Model objective	Target output	Suitable vs unsuitable
Overview	Focal Taxon	Focal Taxon	Mouflon
Overview	Location	Location	Central Iran
Overview	Scale of Analysis	Spatial extent	3,850 km²
Overview	Scale of Analysis	Spatial resolution	30m
Overview	Scale of Analysis	Temporal extent	2020–2024
Overview	Scale of Analysis	Boundary	Natural
Overview	Biodiversity data	Observation type	Field survey; range map
Overview	Biodiversity data	Response data type	Point occurrence
Overview	Predictors	Predictor types	Topographical, human activity, habitat
Overview	Hypotheses	Hypotheses	The effectiveness of the InVEST and MaxEnt models in identifying suitable habitats for the mouflon
Overview	Assumptions	Model assumptions	Rather than using a traditional comparative approach, which typically contrasts model performance metrics such as AUC or prediction accuracy, this method emphasizes their complementary application in conservation planning by integrating habitat quality and species distribution outputs.
Overview	Algorithms	Modelling techniques	MaxEnt; InVEST
Overview	Algorithms	Model complexity	The Receiver Operating Characteristic (ROC) curve was used to assess the InVEST model performance, while the Getis-Ord Gi* hotspot analysis was applied to both models
Overview	Workflow	Model workflow	The maximum entropy (MaxEnt) and Integrated Valuation of Ecosystem Services and Tradeoffs (InVEST) models were employed to assess the mouflon (*Ovis gmelini*)

			habitat in central Iran. This study aims to investigate the complementary role of these models based on their distinct input requirements and analytical approaches- MaxEnt focusing on species occurrence and environmental variables, and InVEST emphasizing land-use data, habitat quality, and anthropogenic threats- in the detection and management of suitable and sensitive habitats. The models' performance was evaluated using the Area Under the Curve (AUC) of the Receiver Operating Characteristic (ROC) curve and the Boyce Index (BI). The spatial distribution of suitable habitats was then analyzed applying a hot spot analysis using the Getis-Ord Gi* statistic on both models' outputs. The results indicated that the MaxEnt model was more effective at recognizing suitable habitats within protected areas (AUC = 0.937, BI = 0.965), while the InVEST model excels in identifying potential suitable habitats for conservation activities and migration corridors in the surrounding, unprotected landscapes. The hotspot analysis, applied to the spatial outputs of both the MaxEnt and InVEST models, revealed that areas with high habitat quality and high suitability overlapped in some core habitats, but distinct spatial differences were also observed in their distribution patterns. To enhance the conservation network and identify ecological corridors, particularly under data-poor conditions, the InVEST model offers valuable guidance for conservation planning. It highlights priority habitats based on habitat quality and anthropogenic pressure layers, and supports the assessment of connectivity among fragmented areas by identifying zones that maintain or improve habitat continuity. However, the complementary role of both models in providing a more comprehensive understanding for management actions is critical. These findings are especially important for the conservation of wide-ranging species such as the mouflon and other ungulates that inhabit both protected and unprotected landscapes.
Overview	Software	Software	To evaluate and quantify mouflon habitat quality, we used the InVEST v.3.4.4 Habitat Quality model, and habitat suitability was predicted using the MaxEnt algorithm.
Data	Biodiversity data	Taxon names	*Ovis gmelini*
Data	Biodiversity data	Taxonomic reference system	We followed the taxonomic framework of the Mammal Species of the World (Wilson & Reeder, 2016) and the IUCN Red List to ensure consistency in species nomenclature throughout the study.
Data	Biodiversity data	Ecological level	Species
Data	Biodiversity data	Data sources	Direct observations, footprint patterns, and feces, with the assistance of game wardens
Data	Biodiversity data	Sampling design	Random
Data	Biodiversity data	Sample size	126 presence points
Data	Biodiversity data	Clipping	Iran

Data	Biodiversity data	Scaling	Rasterisation of polygon maps
Data	Biodiversity data	Cleaning	Moran’s I
Data	Data partitioning	Training data	75% of the occurrence data were used for training
Data	Data partitioning	Validation data	Subsample method
Data	Data partitioning	Test data	30% of the occurrence data were used for testing
Data	Predictor variables	Predictor variables	Environmental layers (30m resolution)
Data	Predictor variables	Data sources	DEM from the National Cartographic Center of Iran, Human activity variables were extracted from 1:50,000 topographic maps, Habitat-related variables obtained from the Forests, Rangelands and Watershed Management Organization
Data	Predictor variables	Spatial extent	3,850 km²
Data	Predictor variables	Spatial resolution	30m
Data	Predictor variables	Coordinate reference system	wgs 1984- zone 39 n
Data	Predictor variables	Temporal extent	2020-2024
Model	Variable pre-selection	Variable pre-selection	VIF and contribution of variables
Model	Multicollinearity	Multicollinearity	VIF
Model	Model settings	Model settings (fitting)	MaxEnt: no settings provided; InVEST: no settings provided
Model	Model estimates	Parameter uncertainty	Replicated runs
Model	Model estimates	Variable importance	Percent contribution
Model	Model selection - model averaging - ensembles	Model selection	Auto feature
Model	Analysis and Correction of non-independence	Spatial autocorrelation	Moran’s I test
Model	Analysis and Correction of non-independence	Nested data	Clustered sampling
Model	Threshold selection	Threshold selection	10th percentile threshold
Assessment	Performance statistics	Performance on training data	AIC; AUC
Assessment	Performance statistics	Performance on validation data	Cross-validation
Assessment	Performance statistics	Performance on test data	AIC; AUC
Assessment	Plausibility check	Response shapes	Response curves
Assessment	Plausibility check	Expert judgement	Selecting variables, value of invest
Prediction	Prediction output	Prediction unit	30 m

Prediction	Prediction output	Post-processing	Clipping by study area, reclassification, mapping, habitat quality assessment, overlaying with threats
Prediction	Uncertainty quantification	Algorithmic uncertainty	The assumption that presence data are representative of the species' realized niche. Use of a background sample instead of true absences. Sensitivity to sampling bias. Feature classes and regularization settings affecting model complexity.
Prediction	Uncertainty quantification	Input data uncertainty	GPS error, Limited sample size; Inaccurate land cover maps
Prediction	Uncertainty quantification	Parameter uncertainty	Regularization multiplier, Feature classes (L, Q, H, P, T), Number of background points, Validation partitioning (k-folds, spatial blocks)
