# Supplementary material for: Habitat suitability assessment of mouflon applying a comparative approach on expert-based and species distribution models
Source: PLoS One. 2026 Sep 21;21(9):e0353063. doi: 10.1371/journal.pone.0353063 (PMC13592577; doi:10.1371/journal.pone.0353063)
Supplement: Supplementary file 4 [file pone.0353063.s004.pdf]

Supporting information

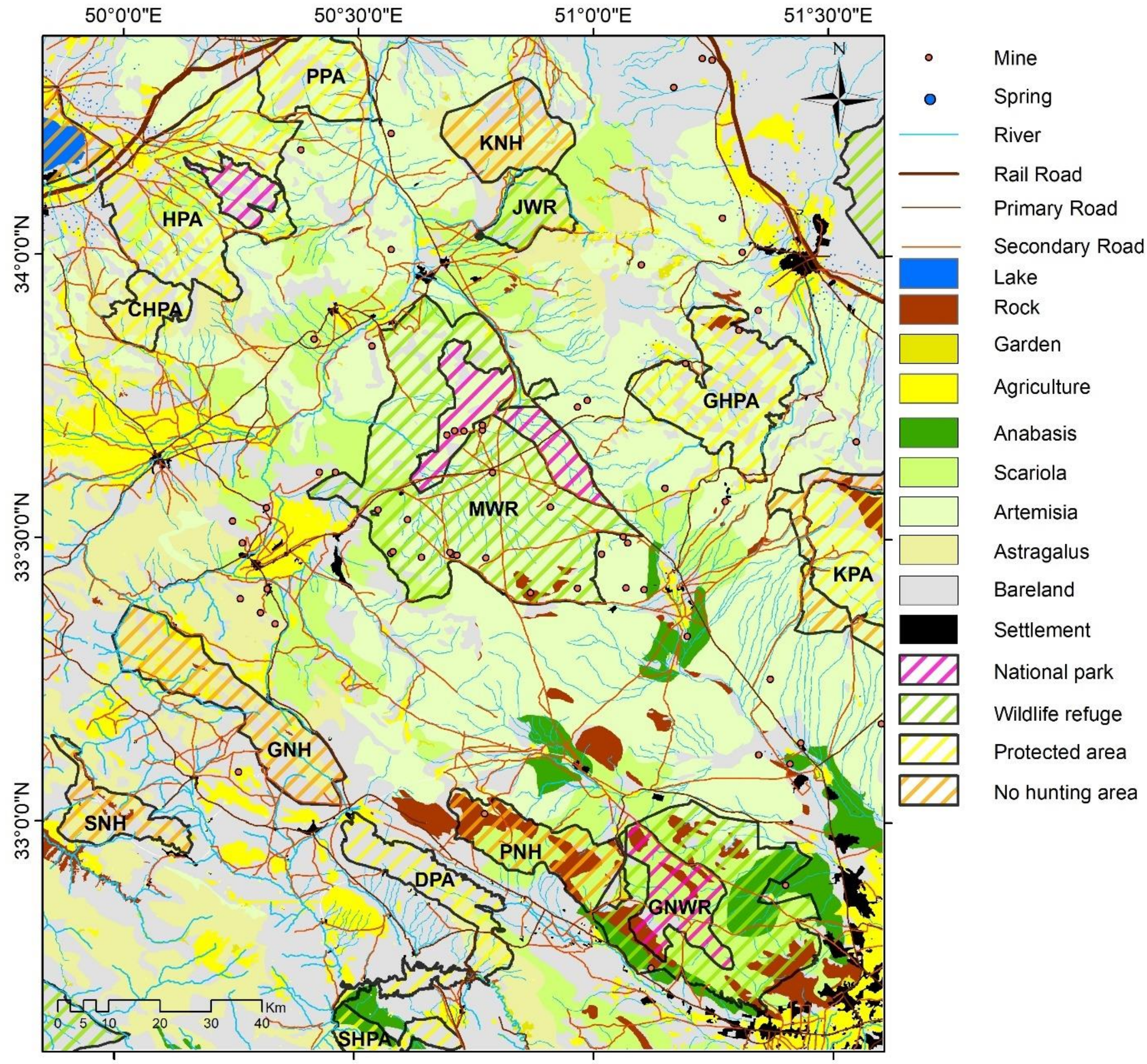

S1 Fig. The land use and land cover map of the study area based on data from topography map (1/50000) obtained from FRWO and DoE. Land uses were aggregated into 17 different main categories corresponding to habitat types.
