# Supplementary material for: Habitat suitability assessment of mouflon applying a comparative approach on expert-based and species distribution models
Source: PLoS One. 2026 Sep 21;21(9):e0353063. doi: 10.1371/journal.pone.0353063 (PMC13592577; doi:10.1371/journal.pone.0353063)
Supplement: Supplementary file 5 [file pone.0353063.s005.pdf]

Supporting information

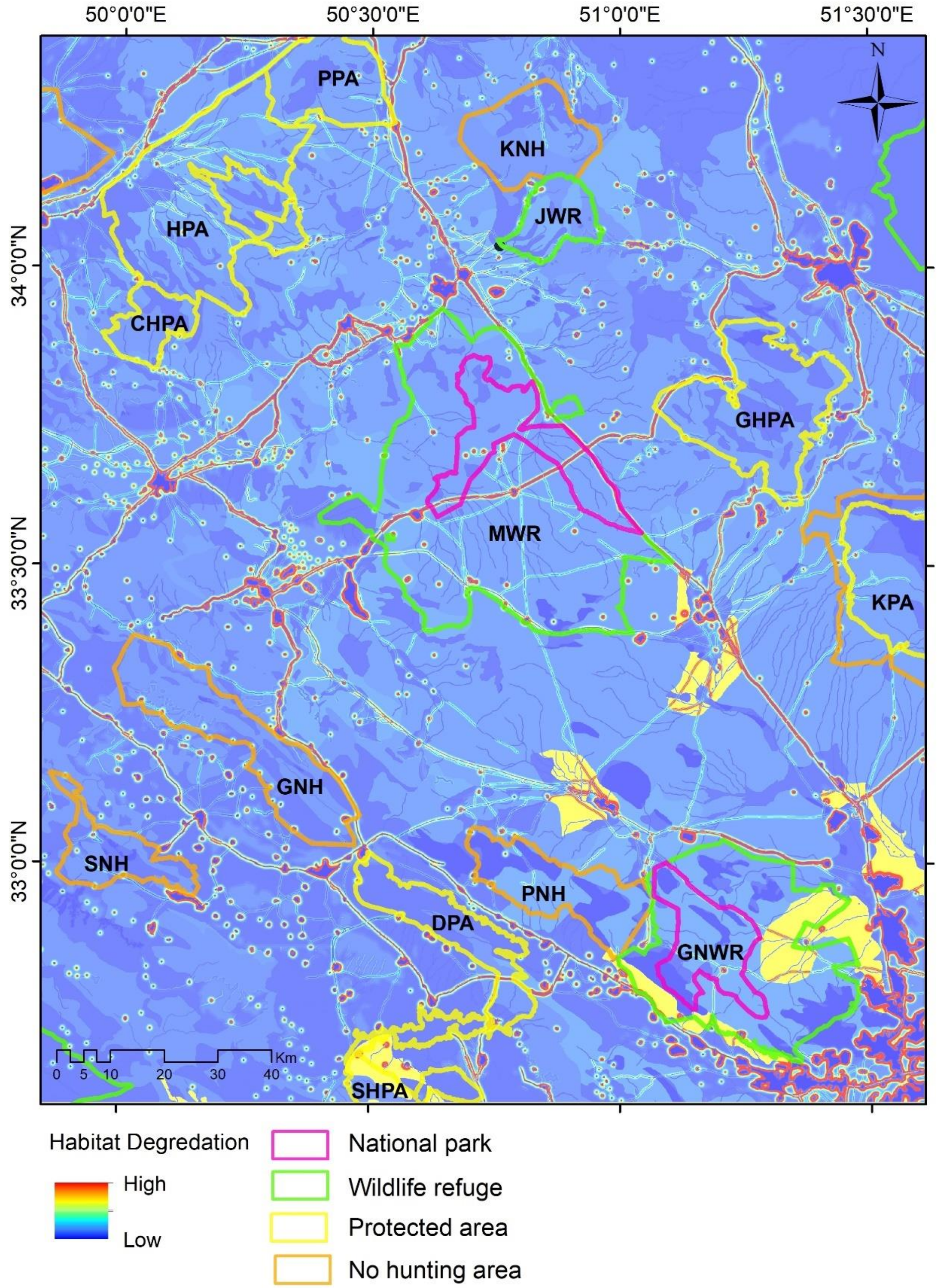

S2 Fig. The degradation map of mouflon in the study area. Habitat quality value ranges between 0 (lowest) and 1 (highest) and habitat degradation is between 0 (minimum) and 0.11 (maximum of threats concentration).
