# Supplementary material for: Habitat suitability assessment of mouflon applying a comparative approach on expert-based and species distribution models
Source: PLoS One. 2026 Sep 21;21(9):e0353063. doi: 10.1371/journal.pone.0353063 (PMC13592577; doi:10.1371/journal.pone.0353063)
Supplement: Supplementary file 6 [file pone.0353063.s006.pdf]

Supporting information

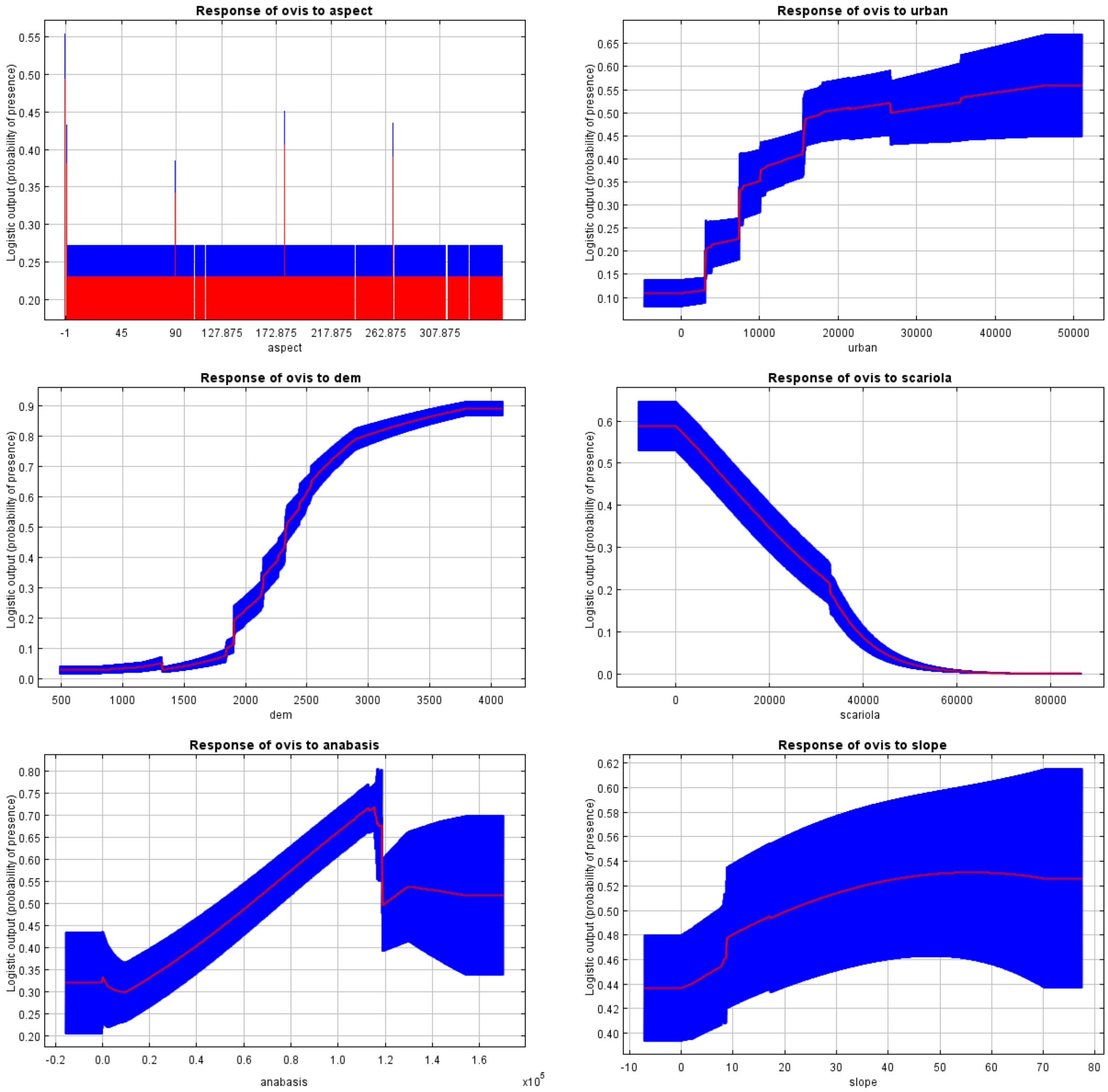

S3 Fig. Response curves showing the effect of key environmental predictors on mouflon habitat suitability in the MaxEnt model.
