# Supplementary material for: Habitat suitability assessment of mouflon applying a comparative approach on expert-based and species distribution models
Source: PLoS One. 2026 Sep 21;21(9):e0353063. doi: 10.1371/journal.pone.0353063 (PMC13592577; doi:10.1371/journal.pone.0353063)
Supplement: Supplementary file 7 [file pone.0353063.s007.pdf]

Supporting information

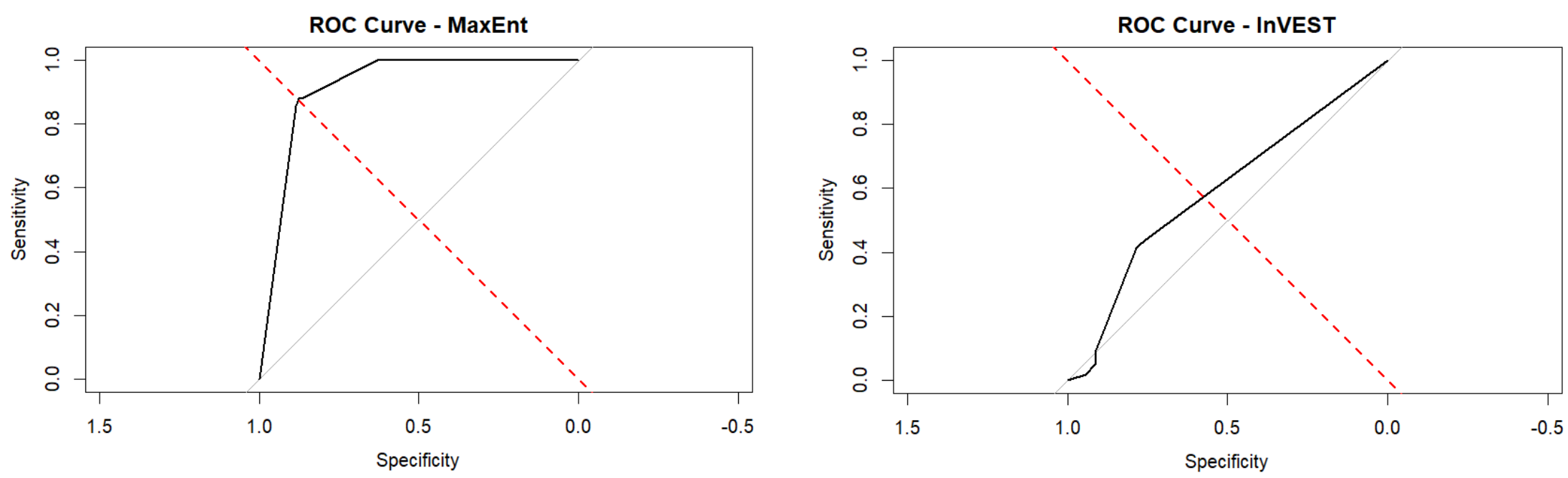

S4 Fig. Performance characteristic diagrams illustrating habitat hot spots. The left panel displays the results from the MaxEnt model, while the right panel presents findings from the InVEST model.
